# Arbovirus Screening in Mosquitoes in Emilia-Romagna (Italy, 2021) and Isolation of Tahyna Virus

**DOI:** 10.1128/spectrum.01587-22

**Published:** 2022-09-27

**Authors:** Mattia Calzolari, Paolo Bonilauri, Annalisa Grisendi, Gastone Dalmonte, Alice Vismarra, Davide Lelli, Chiara Chiapponi, Romeo Bellini, Antonio Lavazza, Michele Dottori

**Affiliations:** a Istituto Zooprofilattico Sperimentale della Lombardia e dell'Emilia Romagna “B. Ubertini” (IZSLER), Brescia, Italy; b Dipartimento di Scienze Medico-Veterinarie, UO di Parassitologia e Malattie Parassitarie, Università di Parma, Parma, Italy; c Centro Agricoltura Ambiente “G.Nicoli,” Crevalcore, Italy; Center for Research and Advanced Studies (CINVESTAV-IPN)

**Keywords:** insect specific flavivirus, mosquito, Tahyna virus, Usutu virus, West Nile virus

## Abstract

Several viruses can be transmitted by mosquitoes. We searched some of these viruses in 20,778 mosquitoes, collected in 95 traps on the plains of Emilia-Romagna (North of Italy) in 2021. We detected West Nile virus (WNV) and Usutu virus (USUV) in pools of *Culex (Cx.) pipiens*. In addition, we detected two insect-specific flaviviruses in three pools of *Aedes (Ae.) caspius* and in two of *Ae. vexans*. Tahyna virus (TAHV) was detected in six pools, three of *Ae. caspius* and three of *Cx. pipiens*, and one isolated strain was obtained from one of the *Ae. caspius* pools. Moreover, we detected TAHV in pools of several mosquito species (*Ae. caspius, Ae. vexans, Ae. albopictus, Anopheles maculipennis s.l.*) collected in the previous year of surveillance. Our data indicate *Ae. caspius* as the species most infected with TAHV in the surveyed area. Together with the likely plasticity of the cycle, we reported strong genome stability of the TAHV, probably linked to a successful adaptation of the virus to its ecological niche. Interestingly, in six pools of *Cx. pipiens* we detected two associated viruses among USUV, WNV, TAHV and all the three viruses in two pools. This result allows us to assume the presence of particular conditions that prompt the circulation of arboviruses, creating the conditions for viral hot spots. While no human diseases related to Tahyna virus were reported in Italy, its detection over the years suggests that it is worth investigating this virus as a potential cause of disease in humans in order to assess its health burden.

**IMPORTANCE** We reported in this work the detection of three Arboviruses (Arthropod-borne viruses) in mosquitoes collected in Emilia-Romagna in 2021. In addition to West Nile and Usutu viruses, which were reported from more than 10 years in the study area, we detected and isolated Tahyna virus (TAHV). We also reported detections of TAHV obtained in previous years of surveillance in different species of mosquitoes. TAHV is the potential causative agent of summer influenza-like diseases and also of meningitis. Even if human cases of disease referable to this virus are not reported in Italy, its relevant presence in mosquitoes suggests investigating the possibility they could.

## INTRODUCTION

Arboviruses (Arthropod-borne viruses), a category that includes viruses from different genera, are defined as viruses transmitted by arthropods. Mosquitoes (1) transmit several arboviruses. Many of them, such as West Nile virus (WNV) and Usutu virus (USUV), belong to the Flavivirus genus, which also includes insect-specific flaviviruses (ISFs). Through entomological surveillance plans, ISFs are being increasingly reported in mosquitoes exclusively and do not seem to be “real” arboviruses, as they do not appear capable of infecting vertebrates (2). Two families of viruses, both belonging to the *Bunyavirales* order, widely represented in arboviruses are *Peribunyaviridae* and *Phenuiviridae*, mainly with the two genera *Orthobunyavirus* and *Phlebovirus*, the latter including viruses often transmitted by sand flies (2, 3).

Several arboviruses are actively monitored for their abundant diffusion, their health relevance, and recent endemization. WNV is one of these viruses, actively surveyed in several areas in Italy, including the Emilia-Romagna region (Northern Italy), with the primary aim of guaranteeing the safety of blood and organ donations (4). In fact, although this virus circulates mainly among birds and mosquitoes, it can infect vertebrates (in particular humans and horses) as dead-end hosts, affecting the central nervous system in a minority of cases and causing severe, sometimes lethal neurological disease (1, 5). USUV is a flavivirus similar to WNV for its ecological characteristics, which also shows neurotrophic capacity, albeit less than WNV (5). The cocirculation of USUV and WNV has previously been recorded in the Emilia-Romagna region through entomological surveillance (6).

Other arboviruses, whose pathogenicity is already proven, may circulate in the environment but are neglected. A paradigmatic example is the Tahyna virus (TAHV), belonging to the family *Peribunyaviridae* and genus *Orthobunyavirus*. Most TAHV infections in humans are unapparent, while, when symptomatic, the infection produces an acute influenza-like disease, mainly in children. Meningitis or other signs of central nervous system involvement have been observed, but no fatalities have been attributed to TAHV (7). However, the World Health Organization (WHO) considers TAHV an important arbovirus agent with respect to public health across Europe (8).

TAHV was the first arbovirus ever isolated in Europe, from *Aedes (Ae.) caspius* and *Ae. vexans* collected in the Tahyna and Krizany villages in East Slovakia (9). The virus was subsequently isolated from several mosquito species and reported in Austria, Czech Republic, Hungary, Slovenia, Serbia, Romania, France, Germany, Norway, and other Eastern European countries such as Estonia, Moldavia, Ukraine, Russia, and Italy (10, 11).

In this study, we made use of the WNV and USUV entomological surveillance established in Emilia Romagna since 2007 (12) by screening mosquitoes caught over a fortnight for a wide range of potentially pathogenic viruses through the application of a panel of specific real-time PCRs and/or traditional PCR protocols and isolation on cell monolayer.

## RESULTS

**FIG 1:**
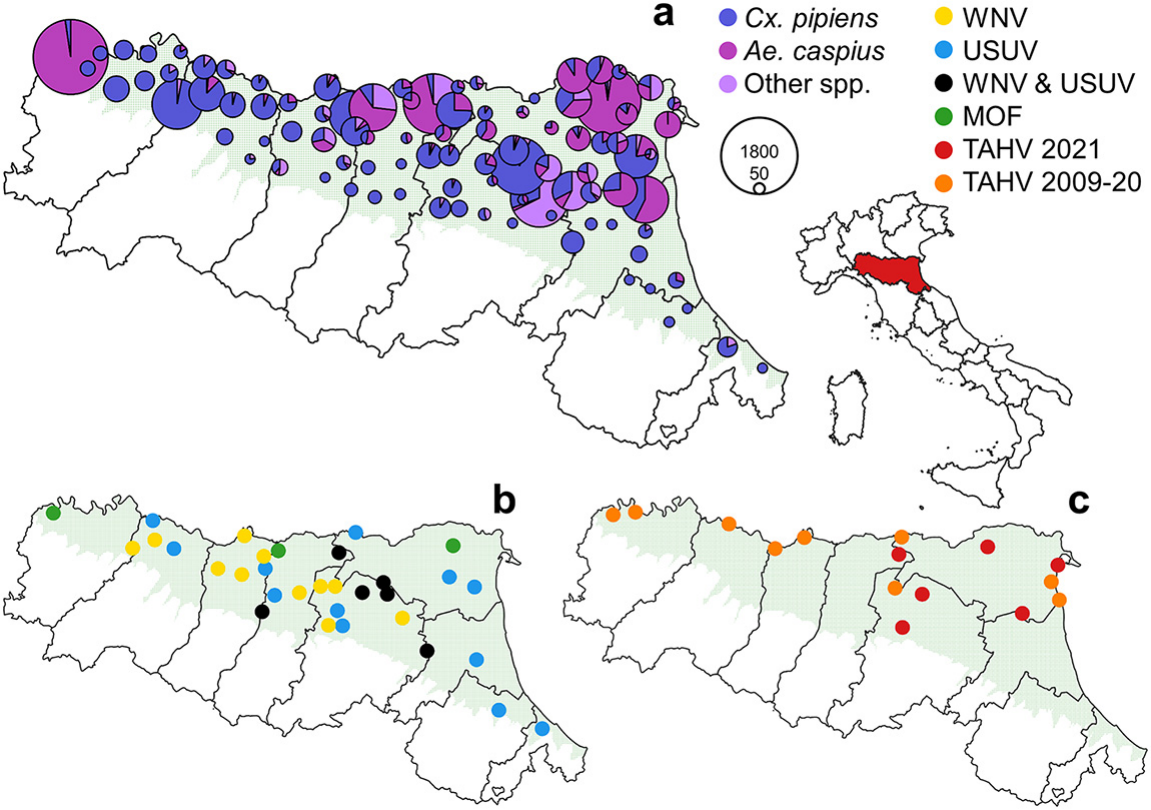
Map of Emilia-Romagna with reference to the location of the region in Italy and surveyed area (green) showing (a) the number of mosquitoes collected at the different sites in 2021 (diameter proportional to the number of mosquitoes collected); (b) the sampling sites of flavivirus positive pools in 2021; (c) the sampling sites of Tahyna virus positive pools in 2021 and in previous years. The maps were produced with the free software QGIS (available at https://www.qgis.org/en/site/index.html, accessed on August 8, 2022).

In the period of August 10 to 19, 2021, we collected 20,778 mosquitoes, belonging to seven species (Fig. 1; Table 1), from the 95 traps of the Emilia-Romagna WND surveillance plan. The most abundantly collected species were *Culex (Cx.) pipiens*, followed by *Ae. caspius* and *Ae. vexans*.

**TABLE 1:** Mosquitoes collected in the 2021 surveillance from August 10 to 19, 2021 with reference to tested specimens and detected viruses

Species	Sampled	%	Tested	Pools	Detected virus
Aedes albopictus	838	4.0	762	29	
*Aedes caspius*	7,586	36.5	7,536	80	3 TAHV^*a*^, 3 ISFs
*Aedes vexans*	1,756	8.5	1,733	22	2 ISFs
*Anopheles maculipennis s.l.*	115	0.6	85	6	
*Anopheles plumbeus*	3	0.0			
*Coquillettidia richiardii*	108	0.5	107	4	
Culex pipiens	10,372	49.9	10,372	114	24 WNV, 21 USUV, 3 TAHV^*b*^
	20,778		20,595	255	

a TAHV isolation achieved from one of these pools.

b Multiple detections: 2 pools WNV/USUV/TAHV; 6 pools WNV/USUV; 1 pool TAHV/USUV.

We tested 20,595 of these mosquitoes in 255 pools. WNV and USUV were detected only in pools of *Cx. pipiens*, 24 pools were WNV-positive and 21 USUV-positive, while eight of these pools, collected at six different sites, tested positive for both viruses (Fig. 1; Table 1). Pan-flavivirus PCR confirmed the results obtained by specific WNV and USUV protocols. Moreover, by applying this PCR, we obtained five sequences ascribable to two ISFs (Fig. S1), of which three were from *Ae. caspius* (GenBank ON124944-ON124946) and two were from *Ae. vexans* (GenBank ON124947-ON124948) (Table 1; Fig. S1). Of the first three sequences, two are identical and showed a p-distance of 0.08 with the third showing a p-distance of 0.07 with the same part of sequence of Marisma mosquito virus (GenBank MF139576). The sequences of the second group are identical between themselves and with the corresponding sequences detected in the Czech Republic (GenBank JN802283) and Italy (GenBank KF801590).

We did not obtain any sequences ascribable to phlebovirus in collected pools using the pan-phlebovirus PCR. By amplicon sequencing of part of the S segment, we detected six pools, three from *Ae. caspius* and three from *Cx. pipiens*, with sequences ascribable to an Orthobunyavirus and all of them were confirmed by Sanger sequencing as Tahyna virus (GenBank ON124938-ON124940 and ON124941-ON124943, respectively). Three of these short sequences (188 nucleotides long) were identical between themselves and identical to 24 sequences already present in GenBank. One of the TAHV-positive *Cx. pipiens* pools was also USUV-positive, other two tested positive for both USUV and WNV; these pools were sampled at three neighboring sites (with an average distance of 26 km), two in the province of Bologna and one in the province of Modena.

We also retrieved the data and sequences of TAHV detections obtained in past surveillance seasons, i.e., a total of 10 sequences (two obtained in 2009, five in 2010, and three in 2020) from four mosquito species (three from *Ae. caspius*, three from *Ae. vexans*, three from *Anopheles maculipennis s.l.* and one from *Ae. albopictus*) (Table 2). All detected strains are grouped in a well-supported branch in the phylogenetic tree obtained by aligning the partial sequences of the small (S) segment of the TAHV virus available in GenBank (Fig. S1).

**TABLE 2:** Tahyna virus detections in previous years of surveillance

Yr	Species	No. mosquitoes	No. pools	NUT3	Municipality	TAHV + pools	GenBank	Collection day
2009	*Aedes caspius*	5,472	67	Ferrara	Comacchio	2	HM068014 HM068015	July 23, August 11
2010		13,724	249	Ferrara	Bondeno	1	JN051146	September 9
2010	*Aedes vexans*	16,106	140	Reggio Emilia	Luzzara	1	JN051149	July 13
				Parma	Roccabianca	2	JN051150 JN051151	July 20
2010	Aedes albopictus	726	43	Reggio Emilia	Brescello	1	JN051147	August 10
2020	*An maculipennis* ^*a*^	474	64	Piacenza	Sarmato	1	ON124935	July 28
				Piacenza	Calendasco	1	ON124936	June 9
				Bologna	San Giovanni in Persiceto	1	ON124937	May 21

a Only species tested in this year.

With the aim of isolating TAHV, we attempted isolation on cell culture from the three *Ae. caspius* positive pools, obtaining the strain 404118 of Tahyna virus, from a pool of 20 *Ae. caspius* sampled on August 10, 2021 in the municipality of Comacchio. Cytopathic effect (CPE) was observed in cell cultures inoculated with the remaining part of a TAHV positive mosquito homogenate. Moreover, reverse transcription PCR (RT-PCR) assays performed on the supernatant of these cultures and pan-orthobunyavirus PCR was positive, demonstrating that the detected viruses had been isolated.

**FIG 2:**
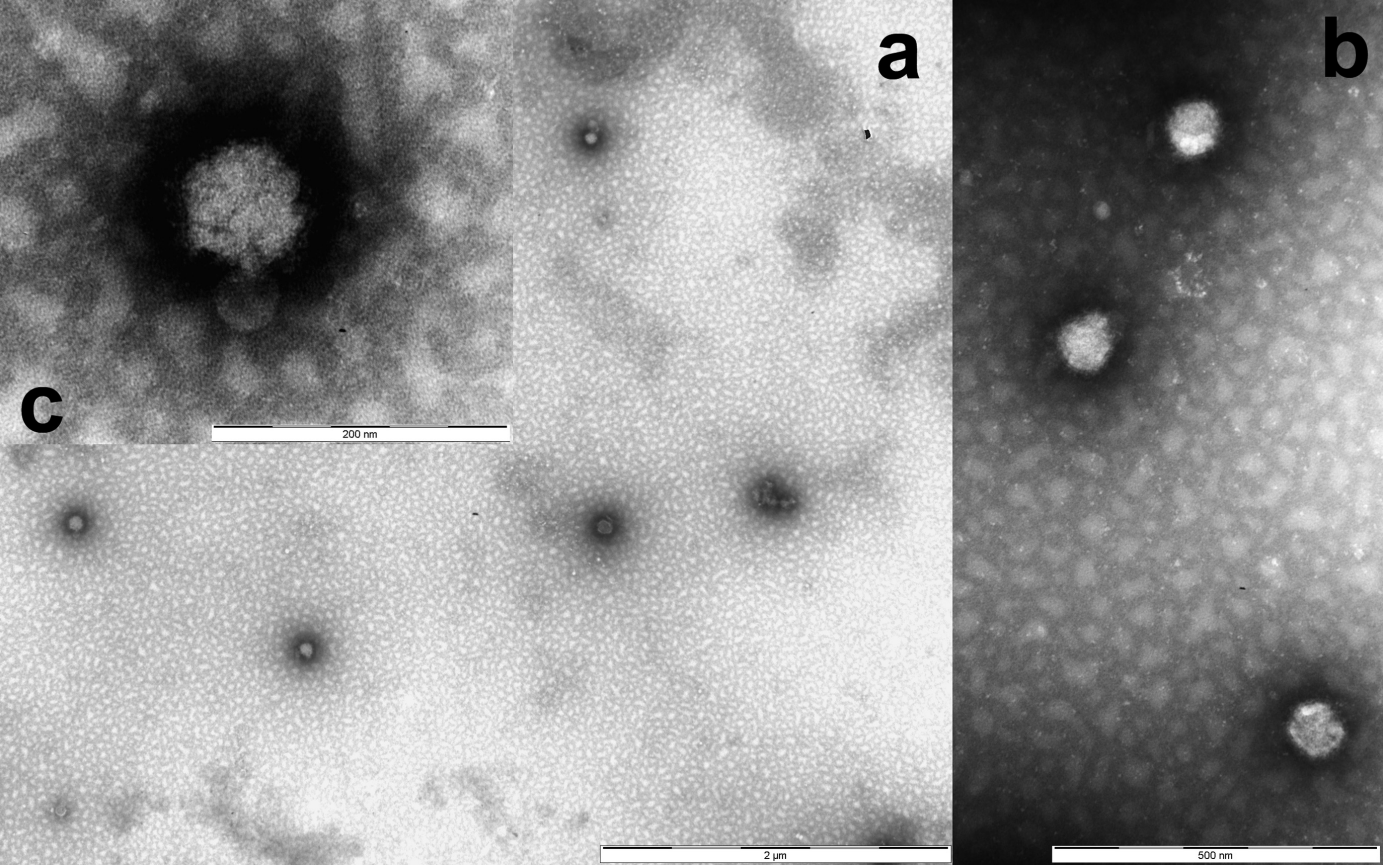
Ultramicrographs of virions observed in the supernatant of VERO cells inoculated with pools of mosquitoes. Roundish particles of 90 to 100 nm with envelopes and tightly packed projections on the surface are shown at low (a), medium (b), and high magnification (c). Negative staining of NaPt 2% (pH 6.8) observed with a TEM FEI Tecnai G2 Spirit Bio-twin.

A preliminary identification of the isolated virus was obtained by examining the supernatants of infected cell cultures with negative staining electron microscopy (nsEM). As shown in Fig. 2, we observed numerous scattered particles morphologically resembling bunyavirus based on typical characteristics, i.e., spherical shape, a diameter of approximately 90 to 100 nm and the presence of tightly packed peplomers (spikes) on the surface.

We obtained the complete sequences of the three genomic segments, which were deposited in GenBank (GenBank L segment, ON156450; M segment, ON156451; S segment, ON156452). Other TAHV complete sequences available in GenBank were referred to strains isolated from mosquitoes, mainly between 1958 and 1968 in former Czechoslovakia. Beyond these, only sequences from strains from France, Austria, and China were available (Table S1). Sometimes the sequences of all three segments of a strain were available. The substitution model selected for the different trees were TN+F+G4 for S segments, TIM2+F+G4 for the L segments, and TIM2+F+I for M segments.

**FIG 3:**
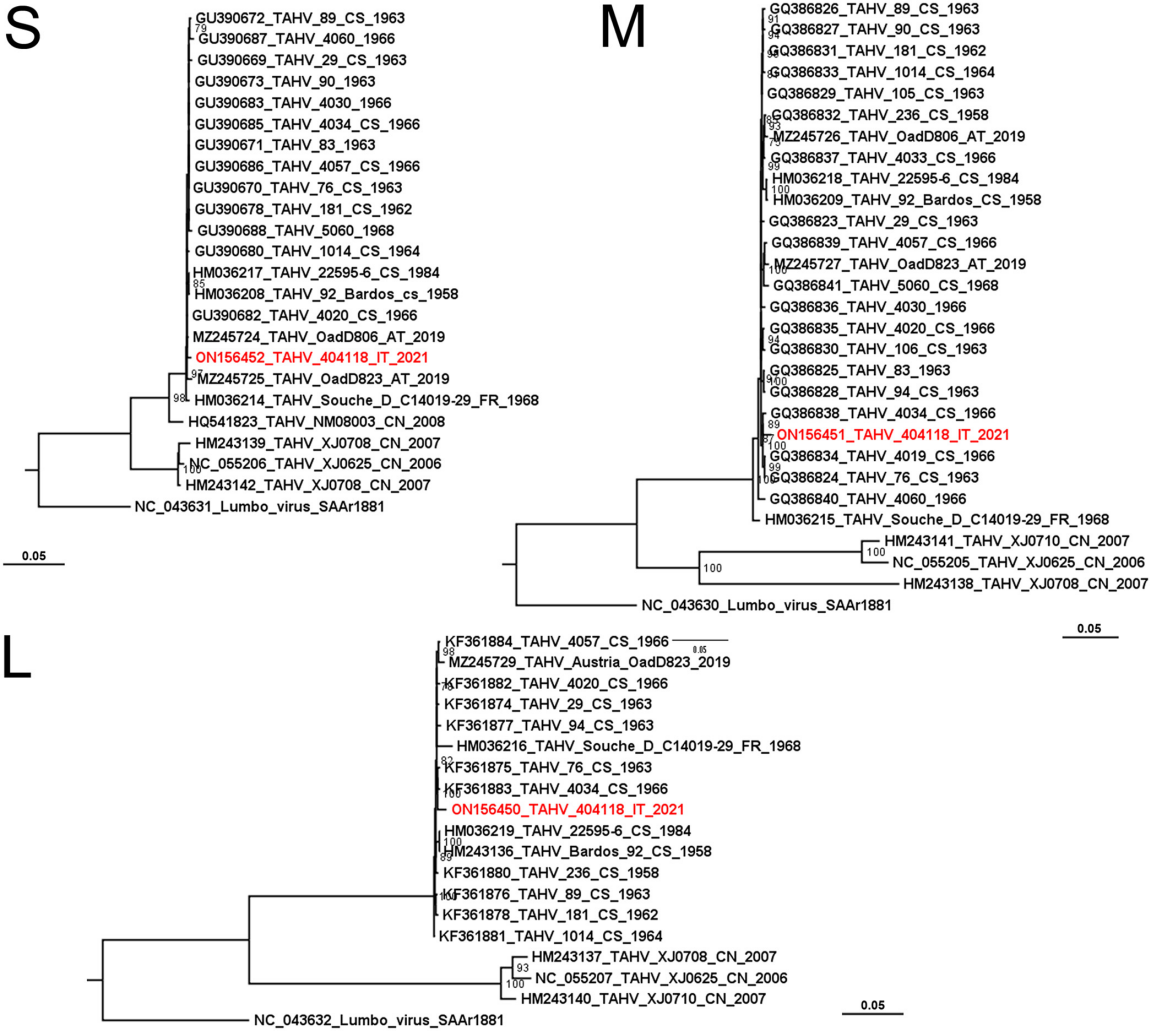
Maximum likelihood trees obtained from the sequences of the three segments of the new isolated Tahyna virus (strain 404118, in red) and homologous sequences retrieved from GenBank. The accession numbers, the country and the year of isolation are reported (CS, former Czechoslovakia; FR, France; CH, China; AT, Austria; IT, Italy). Bootstrap >75 displayed near the respective branch.

Despite the temporal distance with the first isolations, all the sequences obtained in this work are strictly related with other European strains, and more distantly related with Chinese strains (Fig. 3). The average p-distance in the European clade is less than 0.01 for amino acid and nucleotide sequence for all segments.

## DISCUSSION

Screening for detection of flaviviruses, orthobunyaviruses, and phleboviruses in mosquitoes collected over 10 days of sampling provided a snapshot of the arboviruses present in the study area, including one neglected arbovirus.

The detection of WNV and USUV was an expected result, as these viruses have been detected in the study area since 2008 (12). The detection of these two flaviviruses in *Cx. pipiens* pools only confirms this species as the main vector of both viruses in Northern Italy (6, 12).

Less frequent was the detection of sequences related to two ISFs, the first was the Marisma mosquito flavivirus, previously isolated in Spain (13) and detected in Northern Italy, also in Emilia-Romagna (12), always from *Ae. caspius*, as in this study. The second was detected in Italy (14, 15) and the Czech Republic in *Ae. vexans* and *Ae. caspius* (16), and hypothetically ascribable to a sequence integrated in the mosquito genome (14). ISFs have been increasingly reported through entomological surveillance worldwide, demonstrating their diffusion (2). Thus, it was not surprising that two ISFs, which have been already detected in surveyed areas, were recovered in this study. Indeed, their presence does not seem to be a health issue because they seem to be exclusive to mosquitoes (14). However, the characterization of their cycle and possible interaction with pathogenic flaviviruses deserve more experimental study.

Contrary to what was observed for flaviviruses, we did not detect the presence of phleboviruses in the sampled mosquitoes. While sand flies transmit most phleboviruses, several are transmitted by mosquitoes; the most relevant is the Rift Valley Fever virus but also the lesser known phleboviruses such as the Arumowot virus and the Odrenisrou virus have been isolated from mosquitoes and serologically detected in humans in Africa (17).

An interesting result was the detection of TAHV in six pools of mosquitoes sampled in 2021 and in 10 pools sampled in 2009, 2010, and 2020; this confirmed the continuous presence of the virus in the Emilia-Romagna region (12). In Italy, the virus was first isolated in 1968 from *Aedes* mosquitoes in the province of Gorizia (Friuli Venezia-Giulia region, North-Eastern Italy) (18). Subsequently serological evidence confirmed the presence of the virus in humans (19) and in small mammals (rodents, insectivores, carnivores) (20). The cycle of the virus provides as most relevant vertebrate amplifying hosts, at least in central Europe, the European Brown hare (*Lepus europaeus*) and the wild rabbit (*Oryctolagus cuniculus*), both considered highly susceptible to TAHV because they can develop a sufficiently high and long-lasting viremia that is able to infect vectors (7). In addition, some rodents, insectivores, and domestic animals seem accessional hosts (7). While TAHV were serologically detected in birds in several studies, sometimes with high seroprevalence, as in the Czech Republic (21), early studies excluded a relevant role of birds in sustaining the circulation of the virus (22, 23).

TAHV transmission occurs via infected mosquito bites. According to Labuda (7), the most important TAHV vector is *Ae. vexans*, at least in the Czech Republic. Nonetheless the virus has been detected in other mosquito species: *Ae. cinereus*, *Ae. caspius*, *Ae. cantans*, *Ae. punctor*, *Ae. communis*, *Ae. flavescens*, *Ae. excrucians*, *Culiseta (Cs.) annulata*, *Cx. modestus*, *Cx. pipiens*, and *Anopheles hyrcanus* (24–37). Transovarian transmission was documented in *Ae. vexans* (38) and in *Cs. annulata* (39), while possible overwintering was observed in female *Cx. modestus* (40) and *Cs. annulata* (41).

The obtained results confirm the spread and temporal persistence of the TAHV in a large part of the surveyed area. This result was confirmed by the detection and isolation of the virus in the neighboring region Lombardy (42). Furthermore, TAHV positive pools are likely underestimated, as the pan-orthobunyavirus protocol does not perform as well as a specific PCR in terms of sensitivity. In the present work, we detected the virus in several species of mosquitoes, mainly in *Aedes* species, *Ae. caspius* and *Ae. vexans*, and in a pool of *Ae. albopictus*, but also in mosquitoes of the Maculipennis complex and in *Cx. pipiens*. It is worth noting that the 2021 results derived from a systematic and broad approach involving different mosquito species. In previous years, the search was performed only on subsamples representing selected species of mosquitoes. Although detection in a species cannot be considered proof of its vector competence, these results could be explained assuming a wide ecological plasticity of the virus, i.e., the capacity of the virus to be transmitted by different mosquito vectors and infect different hosts in different habitats and eco-geographical areas. The mosquito with the highest incidence of TAHV positive pools in the surveyed area was *Ae. caspius* and, in fact, isolation was achieved from a pool of this mosquito. This indicates *Ae. caspius*—a mosquito much more widespread than *Ae. vexans* in Northern Italy—as the main potential TAHV vector in the studied area.

We detected TAHV in three pools of mosquitoes of the Maculipennis complex in 2020, a season in which all mosquitoes of the complex collected in the framework of the WNV surveillance were tested. TAHV was previously detected from *Anopheles hyrcanus* from the field (28), but not from mosquitoes of the Maculipennis complex.

In the past, TAHV has also been isolated from *Cx. pipiens* (32). In the present work, we detected the presence of the virus in three pools of this mosquito, two of which also tested positive for WNV and USUV. Given the marked ornithophily of *Cx. pipiens*, this result suggests the importance of better investigating the possible role of birds in the transmission of the virus. Furthermore, the presence of three different arboviruses (USUV, WNV, TAHV) in two pools of *Cx. pipiens* highlights the presence of hot spots of arboviral transmission, in which these viruses circulate simultaneously. This hypothesis is supported by the origin of these pools, sampled in two neighboring sites in the area with the most intense WNV circulation (43). This is a surprising finding, considering that their cycles involve different animals and vectors, and suggests the existence of particular environmental conditions capable of prompting the circulation of ecologically different viruses.

Interestingly, we also detected the virus in a pool of *Ae. albopictus*, and this might indicate Tiger mosquito competence for TAHV, even if this species had been previously tested for TAHV vector competence, obtaining low vectorial capacity and no vertical transmission (44). Further experimental work on vectorial competence of *Ae. albopictus* and other species will help to characterize the cycle of TAHV.

The obtained TAHV genome allowed evaluating the relationship of the virus isolated in Italy and the previously isolated strains. All the available genomes were obtained from strains isolated from mosquitoes, mainly in former Czechoslovakia from 1958 to 1964. The vast majority of strains were isolated from *Ae. vexans*, but also other species were represented and the Italian strain was isolated from *Ae. caspius*, this observation seems to confirm the ability of TAHV to exploit different mosquitoes as vectors. Phylogenetic analysis based on the complete sequences of the segments grouped all European strains, characterized by a highly conserved genome, in a well-supported clade, while the Chinese isolates (45) clustered in a different branch. The high rate of identity recorded with strains isolated more than 50 years ago, a significant time for viral evolution, confirmed the genomic stability of this virus (46, 47), likely linked to a high adaptation level of TAHV in its ecological niche.

In Italy, no human cases of TAHV have ever been reported with certainty; however, the spread of TAHV described in this work strongly suggests that TAHV can be considered a possible aetiological agent in cases of summer influenza-like diseases. This virus is also the causative agent of meningitis or other diseases characterized by neurological signs and involvement of the central nervous system, which often remain undiagnosed. Serological presence of the virus was recorded in early studies in different regions of Italy, also with high prevalence (19). Therefore, even considering the limit of specificity of serological tests due to possible cross-reactivity with other orthobunyaviruses, a sero-epidemiological investigation for TAHV in the human populations living in monitored areas should be launched to determine the burden of the virus. In addition, the direct and indirect search for neglected arboviruses through targeted surveillance in animals and humans would be desirable to understand the real burden of these viruses, which is likely underestimated. It must be said, however, that not all meningoencephalitis in which the etiology remains unknown will be explained by the detection of TAHV, given that nervous symptoms are a possible sign of many arbovirus infections (48) or an infrequent manifestation of other viral diseases.

Overall, these findings support the need to maintain an extensive entomological surveillance system, organized to detect not only the viruses that are known to be present in the studied areas (WNV for instance), but also for other possible arboviruses of health relevance, through the application of a systematic screening protocol.

## MATERIALS AND METHODS

### Mosquito sampling.

Samples were retrieved from the regional WNV surveillance system in Emilia-Romagna, which makes use of the 95 georeferenced traps operating on the plains of the region (6) (Fig. 1). Mosquitoes were collected overnight by attractive traps baited with carbon dioxide (49) activated from roughly 17:00 h to 9:00 h the next day. Collected mosquitoes were refrigerated immediately (5 ± 3°C), killed by freezing at −20°C, and identified at species level on a chill table the day of collection by morphological keys (50, 51). We tested the mosquitoes sampled from August 10 to 19, 2021, which represent one round of sampling for each trap. Identified mosquitoes were then grouped in species-specific pools according to the site and day of sampling, with a maximum size of 200 individuals per pool. We tested all the pools of *Culex* mosquitoes, while, for other species, we submitted pools with a minimum of five specimens to the analysis.

Together with the results from this survey, in this study we also reported the information, not yet published, relative to the TAHV positive samples recorded in the past years of surveillance. These previous TAHV sequences were obtained by testing part of the mosquitoes of particular species collected during the entomological surveillance in 2009, 2010 (subsample of *Aedes* mosquitoes), and 2020 (Maculipennis complex mosquitoes).

### Arbovirus screening and identification.

Pooled mosquitoes were stored in 2 mL polypropylene cryotubes or 15 mL Falcon vials for more numerous pools (>50 individuals). Two to four 4.3-mm diameter copper plated round balls (Haendler & Natermann Sport GmbH, Münden, Germany) and 1 mL to 4 mL (1 mL per 50 mosquito) of PBS were added to each tube. Samples were ground for 1 min in a vortex mixer, and then centrifuged at 4,000 × *g* for 3 min. Finally, aliquots were collected from the ground samples and submitted to biomolecular analysis. The remaining part of the mosquito homogenate was kept at −80°C until PCR results were obtained. Viral RNA was extracted from the mosquito tissue homogenate in 96 well-plates using the BioSprint 96 One-For-All Vet kit (Qiagen) and the BioSprint 96 workstation (Qiagen) according to the manufacturer’s instructions. The RNA extracted was retrotranscripted using M-MLV reverse transcriptase RNase (H-) (Promega) following the manufacturer’s instructions, in the presence of random hexamers (Roche).

Samples were submitted to specific real-time PCRs for WNV and USUV and generic PCRs followed by the sequencing of obtained amplicons for flaviviruses, orthobunyaviruses and phleboviruses (52–57) (Table 3).

**TABLE 3:** Biomolecular protocols with references and primer sequences used for arbovirus screening

Viruses	Protocols	Primer Fr	Primer R	Probe	Ref
WNV	RT^*a*^	WN10533-10552 AAGTTGAGTAGACGGTGCTG	WN10625-10606 AGACGGTTCTGAGGGCTTAC	WN10560-10579 CTCAACCCCAGGAGGACTGG	52
WNV/USUV	RT	WN-LCV-F1 GTGATCCATGTAAGCCCTCAGAA	WN-LCV-R1 GTCTGACATTGGGCTTTGAAGTTA	WN-LCV-S1 AGGACCCCACATGTT	53
				WN-LCV-S2 AGGACCCCACGTGCT	
		USU-F ACGGCCCAAGCGAACAGAC	USU-R2 GGCTTGGGCCGCACCTAA	USU-S CGAACTGTTCGTGGAAGG	
USUV	RT	USU-F -AAAAATGTACGCGGATGACACA	USU-R -TTTGGCCTCGTTGTCAAGATC	USU-P -CGGCTGGGACACCCGGATAACC	54
Pan-flavivirus	TS^*b*^	MAMD AACATGATGGGRAARAGRGARAA	cFD2 GTGTCCCAGCCGGCGGTGTCATCAGC		55
		FS 778 AARGGHAGYMCDGCHATHTGGT			
Pan-orthobunyavirus	TS	BCS82C ATGACTGAGTTGGAGTTTCATGATGTCGC	BCS332V TGTTCCTGTTGCCAGGAAAAT		56
Pan-phlebovirus	TS	Phlebo forward 1 TTTGCTTATCAAGGATTTGATGC	Phlebo reverse TCAATCAGTCCAGCAAAGCTGGGATGCATCAT		57
		Phlebo forward 2 TTTGCTTATCAAGGATTTGACC			

a RT, real-time PCR.

b TS, traditional PCR and sequencing.

### Virus isolation and cell culture purification.

Virus isolation was attempted from the remaining part of the mosquito homogenates of positive pools stored at −80°C. Samples were inoculated in a confluent monolayer of VERO cells (African green monkey kidney cells, cell culture biobank of IZSLER, code BSCL86), incubated at 37°C with 5% CO2 and observed daily for 7 days to observe the development of CPE. In the absence of CPE, the cryolysates were subcultured twice into fresh monolayers. We used a primate cell line in the attempts to isolate potentially pathogen viruses.

### Electron microscopy.

The supernatant fluids from cell cultures showing CPEs were subjected to nsEM using the Airfuge method (58). Supernatants were ultracentrifuged (Airfuge, Beckman Coulter Inc. Life Sciences, Indianapolis, IN, USA) for 15 min at 82,000 × *g* using a rotor holding six 175-μL test tubes in which specific adapters for 3-mm carbon-coated Formvar copper grids were placed. The grids were then stained using 2% sodium phosphotungstate (pH 6.8) for 1.5 min and observed under a Tecnai G2 Spirit Biotwin transmission electron microscope (Thermo Fisher, FEI, Hillsboro, OR, USA) at 20,500 to 43,000 × *g* for at least 15 min before being considered negative. Attempts to identify the observed viral particles were based on their morphological characteristics.

### Sequencing and phylogenetic analysis.

Fragments amplified by RT-PCR were sequenced by an automated fluorescence-based technique following the manufacturer’s instructions (ABI-PRISM 3130 Genetic Analyzer).

Whole-genome sequencing was performed from RNA extracted from the cell culture isolates using the MiSeq platform (Illumina, San Diego, CA, USA). Sequencing libraries were made with the Illumina TruSeq RNA Library Preparation kit v 2 according to the manufacturer’s instructions. The full-length genome of sample Tahyna virus 404118 was obtained by de-novo assembling of reads by CLC Genomic Workbench v.11 (Qiagen, Milan, Italy). The sequences were identified by BLAST search in GenBank.

Phylogenetic analysis was performed by retrieving from GenBank (59) the same part of sequences of available viruses. Obtained sequences were aligned by MAFFT (60) using default parameters, alignments were cleared of identical sequences with the ElimDupes tool (https://www.hiv.lanl.gov/content/sequence/elimdupesv2/elimdupes.html). We used MEGAX (61) to obtain amino acid and nucleotide p-distances. Maximum likelihood trees were obtained by alignments using the software IQtree (62). For trees obtained by complete sequences of segments the Lumbo virus was used as an outgroup. We used the IQtree software (62) also to determine the best-fit substitution model. Trees were displayed by FigTree software (available at http://tree.bio.ed.ac.uk/software/figtree/ accessed on 8/8/2022).

### Data availability.

Obtained sequences were deposited in the GenBank database with the following accession numbers: TAHV segments sequences: L ON156450, M ON156451, S ON156452; partial TAHV sequences: ON124935, ON124936, ON124937, ON124938, ON124939, ON124940, ON124941, ON124942, ON124943; partial Flavivirus sequences ON124944, ON124945, ON124946, ON124947, ON124948.
