# Supplementary material for: Arbovirus Screening in Mosquitoes in Emilia-Romagna (Italy, 2021) and Isolation of Tahyna Virus
Source: Microbiol Spectr. 2022 Sep 27;10(5):e01587-22. doi: 10.1128/spectrum.01587-22 (PMC9602283; doi:10.1128/spectrum.01587-22)
Supplement: Supplemental file 1 — Fig. S1 and Table S1. Download spectrum.01587-22-s0001.pdf, PDF file, 0.4 MB [file spectrum.01587-22-s0001.pdf]

Figure S1. Maximum Likelihood trees obtained by sequences of amplicons of pan-orthobunyavirus (a) and pan-flavivirus (b) PCRs and selected homologous sequences deposited in GenBank (accession numbers reported).

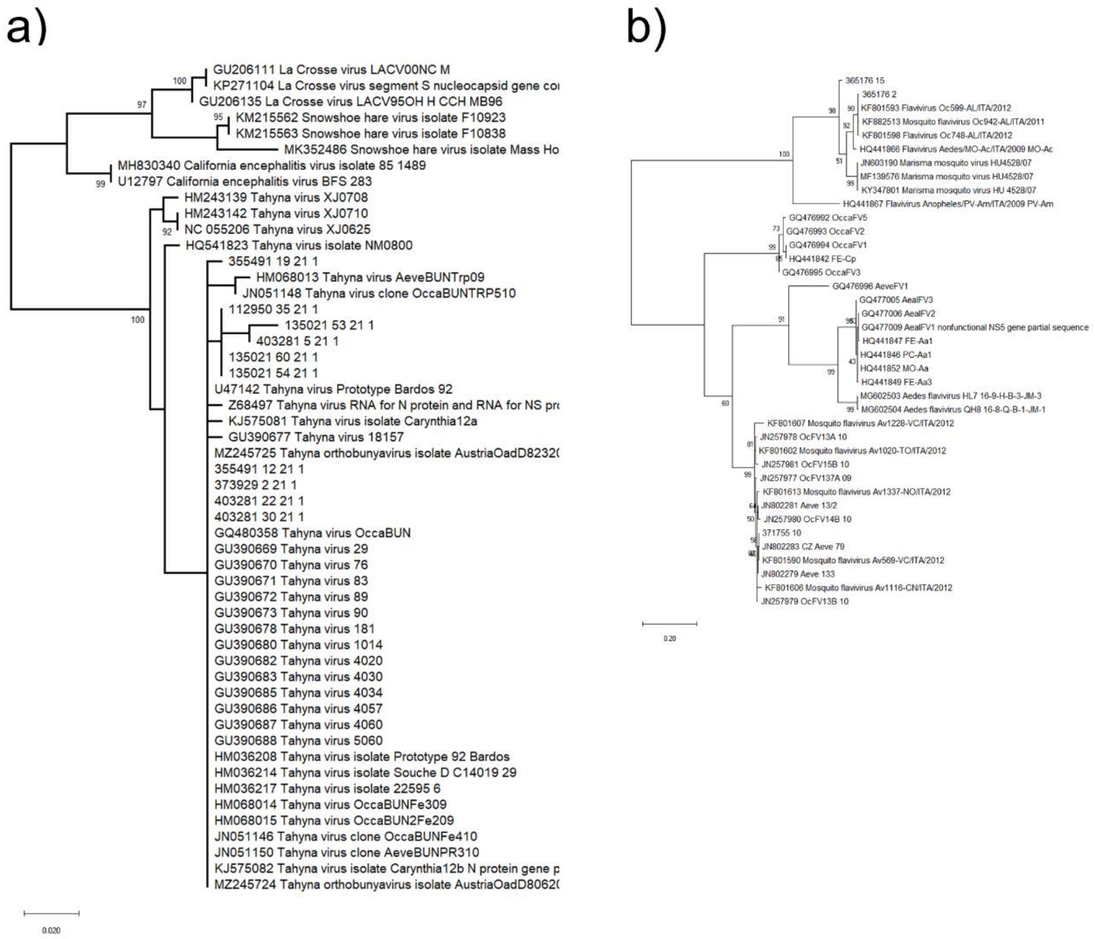

Table S1. Strains used in complete genome phylogenetic analysis with reference to country and year of isolation and GenBank accession numbers.

Strain	Country	Source	Year	L segment	M segment	S segment
Bardos 92	Czechoslovakia	*Aedes caspius Aedes vexans*	1958	HM243136	HM036209	HM036208
236	Czechoslovakia	*Aedes vexans*	1958	KF361880	GQ386832	GU390679*
181	Czechoslovakia	*Aedes vexans*	1962	KF361878	GQ386831	GU390678
29	Czechoslovakia	*Aedes vexans*	1963	KF361874	GQ386823	GU390669
76	Czechoslovakia	*Aedes vexans*	1963	KF361875	GQ386824	GU390670*
89	Czechoslovakia	*Aedes vexans*	1963	KF361876	GQ386826	GU390672
83	Czechoslovakia	*Aedes vexans*	1963		GQ386825	GU390671**
90	Czechoslovakia	*Aedes vexans*	1963		GQ386827	GU390673***
94	Czechoslovakia	*Aedes cantans*	1963	KF361877	GQ386828	GU390674**
105	Czechoslovakia	*Aedes vexans*	1963		GQ386829	GU390675*
106	Czechoslovakia	*Aedes vexans*	1963		GQ386830	GU390676*
1014	Czechoslovakia	*Aedes vexans*	1964	KF361881	GQ386833	GU390680
4019	Czechoslovakia	*Aedes vexans*	1966		GQ386834	GU390681***
4020	Czechoslovakia	*Aedes vexans*	1966	KF361882	GQ386835	GU390682
4030	Czechoslovakia	*Aedes vexans*	1966		GQ386836	GU390683
4033	Czechoslovakia	*Aedes vexans*	1966		GQ386837	GU390684*
4034	Czechoslovakia	*Aedes vexans*	1966	KF361883	GQ386838	GU390685
4057	Czechoslovakia	*Aedes vexans*	1966	KF361884	GQ386839	GU390686
4060	Czechoslovakia	*Aedes vexans*	1966		GQ386840	GU390687
5060	Czechoslovakia	*Aedes vexans*	1968		GQ386841	GU390688
Souche D C14019-29	France	Mosquitoes	1968	HM036216	HM036215	HM036214
22595-6	Czechoslovakia	Mosquitoes	1984	HM036219	HM036218	HM036217
XJ0625	China	*Culex sp.*	2006	NC055207	NC_55205	NC_55206
XJ0708	China	*Aedes vexans*	2007	HM243137	HM243138	HM243139

XJ0710	China	*Aedes vexans*	2007	HM243140	HM243141	HM243142
NM08003	China	*Aedes dorsalis*	2008			HQ541823
Austria/OadD823/2019	Austria	*Aedes vexans*	2019	MZ245729	MZ245726	MZ245725
Austria/OadD806/2019	Austria	*Aedes vexans*	2019	MZ245728	MZ245727	MZ245724
404118	Italy	*Aedes caspius*	2021	ON156450	ON156451	ON156452

* ** *** Identical sequences
